
JOURNAL INFORMATION
==============================
NLM Title Abbreviation: J Med Radiat Sci
Journal ID: J Med Radiat Sci
NLM Journal ID: 101620352
Journal ID: JMRS

Journal of Medical Radiation Sciences

ISSN: 2051-3895
EISSN: 2051-3909
Publisher: Wiley

ARTICLE INFORMATION
==============================
PMCID: PMC11474797
DOI: 10.1002/jmrs.778
Article ID: JMRS778
Article version: 1
Subjects: Issue Information, Issue Information

Issue Information

Electronic publication date: 2024 Mar 7
Print publication date: 2024 Mar
Volume: 71
Issue: 1
First page: 1
Last page: 2
Copyright: © 2024 The Authors. Journal of Medical Radiation Sciences published by John Wiley & Sons Australia, Ltd on behalf of Australian Society of Medical Imaging and Radiation Therapy and New Zealand Institute of Medical Radiation Technology.
License: This is an open access article under the terms of the http://creativecommons.org/licenses/by/4.0/ License, which permits use, distribution and reproduction in any medium, provided the original work is properly cited.
License URL: https://creativecommons.org/licenses/by/4.0/

Figure count: 0
Table count: 0
Page count: 4

source-schema-version-number: 2.0
cover-date: March 2024
details-of-publishers-convertor: Converter:WILEY_ML3GV2_TO_JATSPMC version:6.4.9 mode:remove_FC converted:15.10.2024

==============================

Volume 71, Issue 1 March 2024

### Journal of Medical Radiation Sciences

Open Access

Clinical preparedness
Liver SBRT without fiducial guidance
Value of radiographer commenting
The V.A.G.I.N.A method

WILEY

### Journal of
### Medical Radiation Sciences

Official Publication of the Australian Society of Medical Imaging and Radiation Therapy and New Zealand Institute of Medical Radiation Technology

**Editor-in-Chief**

Cherry Agustin, *The Crown Princess Mary Cancer Centre Westmead, and Blacktown Cancer and Haematology Centre Sydney, Australia (email: Cherry.Agustin@newcastle.edu.au)*

**Deputy Editors**

**Medical imaging**

Karen Dobeli, *Brisbane, Australia*
*Royal Brisbane and Women's Hospital*

**Radiation therapy**

Paul Kane, *Wellington, New Zealand*
*University of Otago*

**Associate Editors**

Linda Bell, *Sydney, Australia*
Elizabeth Brown, *Brisbane, Australia*
Jillian Clarke, *Sydney, Australia*
James Crowhurst, *Brisbane, Australia*
Karen Dobeli, *Brisbane, Australia*
Gay Dungey, *Wellington, New Zealand*
Rhys Fitzgerald, *Bundaberg, Australia*
James Hayes, *Christchurch, New Zealand*
Peter Kench, *Sydney, Australia*
Kellie Knight, *Florida, USA*
Stephen Knight, *Brisbane, Australia*
Michael Neep, *Brisbane, Australia*
Don Nocum, *Sydney, Australia*
Dean Paterson, *Wellington, New Zealand*
Daniel Pham, *Palo Alto, USA*
Ann Poulos, *Sydney, Australia*
Robba Rai, *Sydney, Australia*
Tristan Reddan, *Brisbane, Australia*
Warren Reed, *Sydney, Australia*
Kelly Spuur, *Wagga Wagga, Australia*
Zhonghua Sun, *Perth, Australia*
Andrea Thompson, *Auckland, New Zealand*
Nick Woznitza, *London, United Kingdom*

**Biostatistics Editor**

James Stanley, *Wellington, New Zealand*

**Editorial Review Board**

Patrick Brennan, *Sydney, Australia*
Rob Davidson, *Canberra, Australia*
Georgia Halkett, *Perth, Australia*
Sarah Lewis, *Sydney, Australia*

**International Advisory Panel**

Kamarul Amin Abdullah, *Malaysia*
Michelle Leech, *Dublin, Ireland*
Nicole Harnett, *Toronto, Canada*
Paul Lockwood, *Canterbury, United Kingdom*
Eric Pei Ping Pang, *Singapore*
Wilfred CG Peh, *Singapore*
Suresh Rana, *Miami, USA*
Ronnie A Sebro, *Florida, USA*
Euclid Seeram, *Burnaby, Canada*
Vincent WC Wu, *Hong Kong SAR, China*
Michael Ying, *Hong Kong SAR, China*

**News and Online Editors**

Darien Montgomerie, *Wellington, New Zealand*
Adam Westerink, *Brisbane, Australia*

---

For submission instructions, subscriptions and all other information, visit wileyonlinelibrary.com/journal/jmrs

**Disclaimer:** The Publisher, Australian Society of Medical Imaging, New Zealand Institute of Medical Radiation Technology and Radiation Therapy and Editors cannot be held responsible for errors or any consequences arising from the use of information contained in this journal; the views and opinions expressed do not necessarily reflect those of the Publisher, Australian Society of Medical Imaging and Radiation Therapy or Editors, neither does the publication of advertisements constitute any endorsement by the Publisher, Australian Society of Medical Imaging and Radiation Therapy or Editors of the products advertised.

Journal of
Medical Radiation Sciences

**Volume 71, Issue 1** **March 2024**

### Contents

**Editorials**

Patient experiences and anxiety related to medical imaging: challenges and potential solutions ............... 3
*Kristy Fakes*

Novel genetic assessments for cancer patients: where does medical imaging stand in the future of personalised medicine? ............... 7
*Sally L. Ayesa*

**Original Articles**

Survey of maternal anxiety and perceptions towards foetal MRI and pre-scan education ............... 10
*Stephanie Plunkett, Karen Dobeli, Marita Prior, and Xanthe Tusek*

From planned neck dissection to PET response to circulating tumour DNA: changes in post-treatment assessment of HPV-driven oropharyngeal cancer ............... 21
*Carl M. Post, Kim C. Ohaegbulam, and Timur Mitin*

Optimising image quality in intravenous cerebral cone beam computed tomography ............... 26
*Lisa Broadley, Brendan Erskine, Elissa Marshall, Kyle Ewert, and Bjoern Picker*

Comparison of typical radiation doses and risks using an anthropomorphic 'bone fracture' phantom for commonly performed X-ray projections in a 5-year-old ............... 35
*Edel Doyle, Matthew R Dimmock, Kam L Lee, Peter Thomas, and Richard B Bassed*

A comparison of bolus track and test bolus computed tomography pulmonary angiography and the implications on pulmonary and aortic vessel enhancement, effective dose and suboptimal scan rate ...... 44
*Aoife Murphy, and Helen White*

Correlation of magnetic resonance spectroscopy and magnetic resonance imaging with findings of electroencephalography in patients with temporal lobe epilepsy ............... 51
*Ghazaleh Jamalipour Soufi, Ali Hekmat Nia, Parvaneh Hajalikhani, Jafar Mehvari-Habibabadi, and Navid Chit Saz*

How efficient are metal-polymer and dual-metals-polymer non-lead radiation shields? ............... 57
*Zaker Salehi, and Mansour Tayebi Khorami*

Clinical preparedness programme as perceived by first-year diagnostic radiography students in South Africa ............... 63
*Heidi Thomas, and Gerhardus George Visser Koch*

Identifying delays in the outpatient interventional radiology department: a quality improvement study ............... 72
*Jeremy Kilgour, Glenda McLean, Eldho Paul, and Richard Knight*

A simplified low-cost phantom for image quality assessment of dental cone beam computed tomography unit ............... 78
*James Anthony Rabba, Hanis Arina Jaafar, Fatanah Mohamad Suhaimi, Mohd Zubir Mat Jafri, and Noor Diyana Osman*

Effects of body part thickness on low-contrast detail detection and radiation dose during adult chest radiography ... 85
*Sadeq Al-Murshedi, Kholoud Alzyoud, Mohamed Benhalim, Nadi Alresheedi PhD , Stamatia Papathanasiou, and Andrew England*

A multi-centre survey of New Zealand cancer patients' preferences for radiation treatment information ... 91
*Alannah Flockton, Aidan Leong, Daniel Gilfillan, and Peter Larsen*

Situational anxiety in head and neck cancer: Rates, patterns and clinical management interventions in a regional cancer setting ... 100
*Erin Forbes, Kerrie Clover, Sharon Oultram, Chris Wratten, Mahesh Kumar, Minh Thi Tieu, Gregory Carter, Kristen McCarter, Ben Britton, and Amanda L. Baker*

Liver stereotactic body radiation therapy without fiducial or retained ethiodized oil guidance warrants greater than 5 mm planning target volumes ... 110
*Alec Breazeale, Ramtin Rahmani, Kyle Gallagher, and Nima Nabavizadeh*

Improvement in male pelvis magnetic resonance image contouring following radiologist-delivered training ... 114
*Bronwyn Oar, Amy Brown, Glen Newman, Alan Boles, Christopher N. Rumley, Rachel Doyle, John Baines, and Alex Tan*

**Review Articles**

Formalising written preliminary image evaluation by Australian radiographers: a review of practice value ... 123
*Allie Tonks, Justin Varcoe, and Siena Maurici*

Brain metastases from breast cancer using magnetic resonance imaging: A systematic review ... 133
*Mahdi Mohammadi, Sana Mohammadi, Hojatollah Hadizadeh, Mahsa Olfati, Fatemeh Moradi, Ghazal Tanzifi, and Sadegh Ghaderi*

**Original Article**

Diagnostic radiographers' perceptions of professional identity in Johannesburg, Gauteng, South Africa ... 142
*Keleabetswe Mtombeni, Lynne Hazell, and Louisa Mokoena*

**How I do it**

Viscous Aqueous Gel Illustrating Natural Anatomy: The VAGINA method in gynaecological MRI simulation ... 150
*Kate Skehan, Matthew Richardson, Laura M O'Connor, Samuel Dickson, Kate Martin, Geetha Govindarajulu, and Swetha Sridharan*

**Case Report**

To degas or not to degas? The effectiveness of pharmaceuticals in reducing the impact of bowel volume variations during liver SBRT treatment ... 156
*Phoebe Haslam, Elizabeth Brown, Julie Burbery, Catriona Hargrave, and Yoo Young Lee*

**Continuing Professional Development**

Continuing Professional Development - Medical Imaging ... 163
Continuing Professional Development - Radiation Therapy ... 164

ISSN: 2051-3895

© 2024 *Journal of Medical Radiation Sciences published by John Wiley & Sons Australia, Ltd on behalf of Australian Society of Medical Imaging and Radiation Therapy and New Zealand Institute of Medical Radiation Technology*
